# Aggressive Pelvic Angiomyxoma Presenting As Vulvar Swelling in a Young Female: A Radiologic and Pathologic Correlation

**DOI:** 10.7759/cureus.90073

**Published:** 2025-08-14

**Authors:** Quang Dai La, Aiman Baloch, Muhammad Ayub, Sobia Ahmed, Farzana Jaffar, Benazir Gul, Francis Pryor, Pari Gul

**Affiliations:** 1 Medicine, The Innovative STEMagazine 501(c)3, College Station, USA; 2 Biology, Texas A&M University, College Station, USA; 3 Medicine, Mekran Medical College Turbat, Balochistan, PAK; 4 Radiology, Bolan Medical Complex Hospital, Quetta, PAK; 5 Medicine, Lake Erie College of Osteopathic Medicine, Erie, USA; 6 Radiology, Bolan Medical College, Quetta, Karachi, PAK

**Keywords:** aggressive angiomyxoma, contrast-enhanced ct, estrogen receptor–positive tumor, gynecologic oncology, image-guided biopsy, pelvic soft tissue tumor, perineal tumor, vulvar mass

## Abstract

Aggressive angiomyxoma (AA) is a rare mesenchymal tumor that primarily occurs in women of reproductive age and arises in the pelvis and perineum (perineal area). We present the case of a 20-year-old unmarried female from Afghanistan who was sent for assessment of worsening swelling in the left vulvar and perineal regions. Imaging demonstrated a large, minimal enhancement, soft tissue mass in the left hemipelvis, which displaced organs but otherwise demonstrated no invasion. The lesion extended to the ischiorectal fossa and perineum. Owing to the clinical and radiologic findings, aggressive angiomyxoma was provisionally diagnosed, which was confirmed histologically by image-guided biopsy. The patient underwent surgical resection. Given the tumor has a known local recurrence risk, and it stained positive for estrogen and progesterone receptors, long-term follow-up and adjuvant hormonal treatment were recommended. This case highlights the importance of considering aggressive angiomyxoma in the imaging workup of vulvoperineal masses in younger women and the importance of multidisciplinary management, including imaging, to optimize patient outcomes.

## Introduction

Aggressive angiomyxoma (AA) is a rare, slow-growing mesenchymal neoplasm most commonly found in the pelvic and perineal region of women of reproductive age (typically in the third and fourth decades of life) [1-2]. The tumor was first described and titled by Steeper and Rosai in 1983, given its characteristic infiltrative growth pattern, benign appearance, and inclusion in the category of locally aggressive tumors [3]. Between 2016 and 2019, the incidence of AA was 91.46 to 92.90 instances per 100,000 patient-years, while the prevalence ranged from 0.199% to 0.222% [4].

Clinically, AA often presents as a painless, soft, poorly defined vulvoperineal or pelvic mass; this explains why AA is often misdiagnosed clinically for more common benign lesions such as a Bartholin gland cyst, lipoma, or hernia [5]. Bartholin gland cysts are usually superficial, well-demarcated, and located in the posterior vestibule, while AA usually infiltrates more deeply into the pelvic planes with ill-defined margins, leading to misdiagnosis and delay in treatment [5-6]. AA’s slow progression and deep location often lead to patients with significant enlargement of the mass only presenting late for definitive diagnosis [7].

Imaging appearances radiologically are also non-specific, with contrast-enhanced CT usually displaying a hypo- to isodense mass lesion with minimal or no enhancement that displaces but does not invade the surrounding pelvic structures, while MR imaging usually reveals a classic "whorled" or layered internal pattern on T2-weighted images [2,8].

Histopathologically, AA can be recognized by a myxoid stroma and irregularly arranged spindle and stellate cells, as well as a prominent vasculature. It is typical that immunohistochemical studies are positive for vimentin, desmin, estrogen receptors, and progesterone receptors, thus determining its hormone-sensitive nature [2,9].

AA is characterized at the molecular level by chromosomal rearrangements, including at least in the 12q15 region, and overexpression of the gene High Mobility Group AT-Hook 2 (HMGA2). HMGA2 is a gene that encodes a transcriptional regulator with a significant role in tumorigenesis [10]. The detection of HMGA2 overexpression by immunohistochemistry or fluorescence in situ hybridization (FISH) may help in distinguishing AA from histologically similar neoplasms with myxoid changes [11].

We report a case of AA in a 20-year-old unmarried Afghan female patient who presented with progressive growth of left vulvar and ischiorectal space swelling. Imaging showed a large, minimally enhancing mass causing displacement of the uterus, bladder, and rectum, and confirming AA. Diagnosis came from histopathology of an image-guided biopsy. This is an important case, particularly given the age of the patient and the size of the lesion, and illustrates the need to keep AA in the differential diagnosis for vulvoperineal masses even in atypical demographics. 

This paper was presented as a poster at the Fifth International Radiology Resident Forum Conference on October 13th, 2024.

## Case presentation

A 20-year-old, unmarried female with a history of being born in Afghanistan presented to a gynecology clinic with a history of progressive enlargement of the left vulva. She had a history of pelvic surgery. Neither medical nor surgical records were made available that would describe what type of surgery had occurred. The swelling was soft to palpation and was non-pulsatile and non-tender; clinically, it was determined that the swelling was in the left perineal area. 

**Figure 1:**
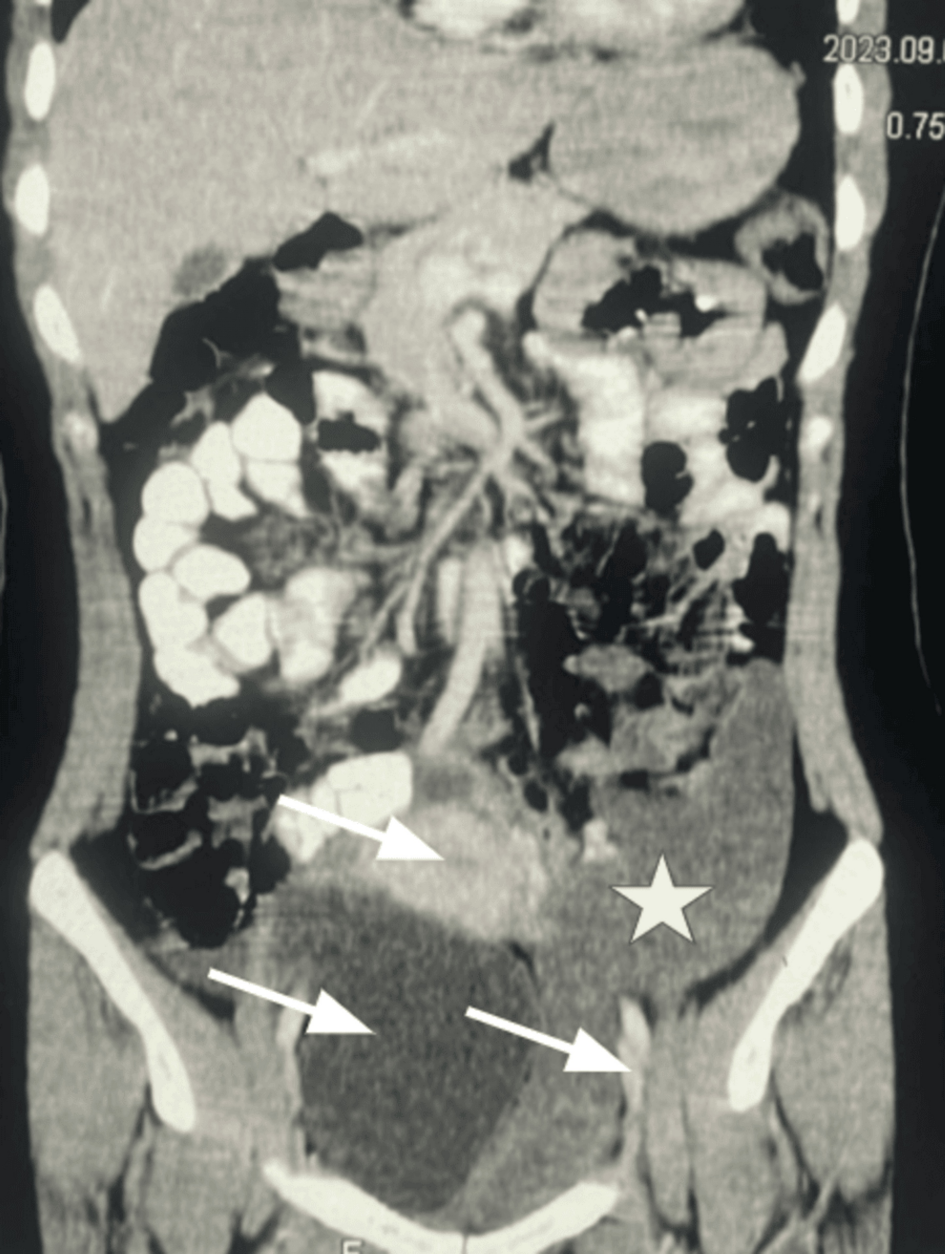
Contrast-enhanced CT of the abdomen showing a large, minimally enhancing soft tissue lesion occupying the left pelvis, with anterior displacement of the uterus and compression of surrounding pelvic structures. Star marks the center of the mass while arrow points to areas of interest.

**Figure 2:**
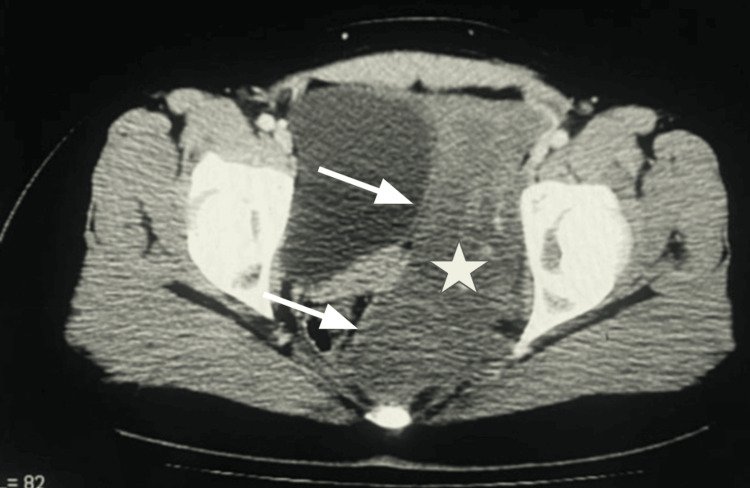
Axial contrast-enhanced CT of the pelvis demonstrating the lesion extending into the left ischiorectal fossa and perineum, without evidence of invasion into the adjacent rectum or bladder. Star highlights the center of the mass. Arrows point to the boundaries of the bladder and rectum.

A contrast-enhanced computed tomography (CT) scan of the abdomen and pelvis showed a large soft tissue attenuation mass with minimal enhancement occupying the left hemipelvis. The mass bulged into the left ischiorectal fossa and extended inferiorly into the perineum (Figures 1, 2). There was considerable compression and displacement of the surrounding pelvic organs (the uterus, urinary bladder, and rectum); however, there was an intact visceral fat plane and no evidence of direct invasion. Given the clinical and radiological appearance, a provisional diagnosis of aggressive angiomyxoma was established, confirmed with histopathology following image-guided biopsy.

## Discussion

This case of a 20-year-old Afghan female with a large, minimally enhancing pelvic mass that extended into the perineum adds to the age demographic of aggressive angiomyxoma (AA), as AA typically occurs in women between 30 and 40 years of age [12]. The patient's age and very large lesion heighten the significance of AA to be included in any differential diagnosis of vulvoperineal masses, even in unusual age groups. 

AA has reported local infiltration rates and recurrence rates between 30-72%, making distant metastasis (<5%) very uncommon [13]. Our evaluation of the imaging characteristics of AA - well-defined, hypoenhancing on CT, and displacement of surrounding tissue - was typical of those demonstrated in the literature describing imaging characteristics overall. 

The recommended treatment is surgical excision with negative margins. The infiltrative nature and lack of awareness of the variable intimate relationship and capsular definition of an AA make complete surgical excision difficult, thus possibly accounting for the high recurrence rates [12]. So, an important consideration is long-term follow-up MRI since recurrences have been reported decades later.

Hormonal therapy is a useful adjunct. Gonadotropin-releasing hormone agonists (GnRH‑a) reduced the size of the tumor before surgery and prevented recurrence after surgery in hormone receptor-positive AA [14]. A case showed that preoperative administration of GnRH decreased tumor burden and made the resection easier, and then after surgery, the use of GnRH-a prevented recurrence in a 28-year-old female patient [15]. Other methods of hormonal therapy, like aromatase inhibitors, tamoxifen, or raloxifene, can be considered, especially if the tumor is recurrent or unresectable.

In this patient’s case, the management of surgical resection confirmed with histopathology was consistent with the best management. The tumor was positive for estrogen and progesterone receptors; therefore, there is a strong indication of adjuvant hormonal therapy to reduce the risk of recurrence. There should be regular, structured long-term follow-up and serial imaging.

This case adds to the literature by describing a very aggressive AA in a significantly younger adult and supports a collaborative, individualized approach of complete surgical excision, adjuvant therapy based on hormones, and long-term monitoring with caution.

## Conclusions

Aggressive angiomyxoma is a locally infiltrative soft tissue tumor that creates diagnostic and management challenges, especially when it is seen outside its usual demographic. This case serves as a reminder that aggressive angiomyxoma should be considered in the differential for perineal and pelvic masses, including in women as young as their early twenties. The slow clinical course and seemingly benign presentation of aggressive angiomyxoma can result in delayed diagnosis. These factors emphasize the need for increased clinical vigilance for atypical presentations of this neoplasm. Complete surgical excision is the optimal management; however, long-term follow-up is important due to the likelihood of local recurrence. If the tumor expresses hormone receptors, adjuvant hormone therapy may be another useful option for reducing the risk of recurrence. Overall, a multidisciplinary approach based on the individual’s clinical context is vital to improving outcomes in patients with this rare tumor.
